# Going virtual: youth attitudes toward and experiences of virtual mental health and substance use services during the COVID-19 pandemic

**DOI:** 10.1186/s12913-021-06321-7

**Published:** 2021-04-14

**Authors:** Lisa D. Hawke, Natasha Y. Sheikhan, Karen MacCon, Joanna Henderson

**Affiliations:** 1 Centre for Addiction and Mental Health, 80 Workman Way, Toronto, Ontario Canada; 2 Department of Psychiatry, University of Toronto, Toronto, Ontario Canada

**Keywords:** Youth, Mental health, Substance use, Virtual services, Telemedicine

## Abstract

**Background:**

During the COVID-19 pandemic, youth mental health and substance use services rapidly moved to virtual modalities to meet social distancing requirements. It is important to understand youth attitudes toward and experience of virtual services.

**Objective:**

This study examined the attitudes toward and experiences of virtual mental health and substance use services among youth drawn from clinical and non-clinical samples.

**Method:**

Four hundred nine youth completed a survey including questions about their attitudes toward and experience of virtual services. The survey included quantitative and open-ended questions on virtual care, as well as a mental health and substance use screener.

**Results:**

The majority of youth with mental health or substance use challenges would be willing to consider individual virtual services, but fewer would consider group virtual services. However, many have not received virtual services. Youth are interested in accessing a wide variety of virtual services and other supportive wellness services. Advantages and disadvantages of virtual services are discussed, including accessibility benefits and technological barriers.

**Discussion:**

As youth mental health and substance use services have rapidly gone virtual during the COVID-19 pandemic, it is essential that we hear the perspectives of youth to promote service utilization among those in need. Diverse, accessible, technologically stable virtual services are required to meet the needs of different youth, possibly with in-person options for some youth. Future research, engaging youth in the research process, is needed to evaluate the efficacy of virtual services to plan for the sustainability of some virtual service gains beyond the pandemic period.

## Highlights

- Youth mental health and substance use services moved rapidly to online service delivery modalities due to the COVID-19 pandemic.
- Understanding youth perspectives on virtual service delivery is essential.
- Most youth are willing to use individual virtual services, but fewer are willing to use group virtual services.
- Virtual services have several advantages, such as accessibility, but also disadvantages, such as technological barriers.
- Diverse, accessible, technologically stable virtual services are required to meet the needs of different youth.

## Background

The COVID-19 pandemic has transformed the way mental health and substance use (MHSU) services are delivered around the world. Services moved rapidly into virtual formats, with little opportunity for thorough planning, to comply with social distancing requirements [1]. Individuals who were previously connected with in-person care have had to rapidly shift in the way they access services, while those who are newly accessing care have had to navigate a new virtual service system to access services for the first time.

Adolescence and emerging adulthood is a key developmental period for the emergence of MHSU challenges [2–4]. In Canada, about one in five youth had MHSU disorders prior to the COVID-19 pandemic [5]. This is a key developmental period, marked by developmental milestones such as progress and transitions in education, labor force integration, occupational development, social relationships, and autonomy [6]. However, public health pandemic guidelines have interrupted many milestones for youth. For example, educational progress and experiences, employment, and social interaction have been disrupted, which may constitute risk factors for emerging or worsening MHSU challenges. Indeed, youth are experiencing mental health challenges during the pandemic [7–12], including heighted symptoms of internalizing mental health challenges [7, 13], post-traumatic stress disorder [10, 13], and general stress and distress [10–12]. Behaviors associated with the maintenance of positive mental and physical health, such as physical activity and sleep, have also been disrupted for many youth [12, 14, 15].

Although the rates of MHSU service use have been increasing in recent years [16], many youth in need do not access services [17, 18]. Research conducted prior to COVID-19 has highlighted many service access barriers, such as long waitlists, uncoordinated services, age-based transition challenges, and stigma [19–23]. System transformation initiatives have been underway to address many of these barriers by creating integrated, youth-friendly service delivery models [24, 25]. However, none of those initiatives foresaw the rapid and systemic shift toward virtual services that occurred with the onset of the COVID-19 pandemic.

Delivering MHSU services virtually is not a new idea. A wide variety of virtual modalities of service delivery have been developed and tested over the past decades [26–28]. Some efficacy has been found, with benefits such as easier accessibility, greater flexibility, lower cost, and reduced stigma [26–28]. While these findings set the stage for transitioning to virtual services with optimism, the urgent nature of COVID-19 pandemic did not provide time to conduct reviews of evidence-based virtual service delivery models and paced implementation according to the implementation science literature [29]. This rapid shift in service modality provides a time-sensitive opportunity to generate new evidence to better understand virtual service delivery [30–32].

The Margaret and Wallace McCain Centre for Child, Youth and Family Mental Health at the Centre for Addiction and Mental Health works from a stakeholder-engaged standpoint to advance research with real-world implications for youth, caregivers, and the service delivery system [33]. Our community mental health partners identified virtual service acceptability among youth during COVID-19 as a priority area of inquiry. Specifically, youth-serving organizations wanted to hear how willing youth are to consider virtual services, what kinds of services they want, what their experience of virtual services has been, and why some youth are not willing to access virtual services. When developing new services, it is essential to understand service users’ experiences of and perspectives on these services [34, 35]. To promote access, services must be designed and delivered in manners that are responsive to service users’ preferences [30, 32].

Given the rapidity with which the transition to virtual models of service delivery occurred due to the physical distancing requirements of the COVID-19 pandemic, without the time required to rigorously follow the evidence base with regard to virtual service design and implementation, it is essential that we examine the success of virtual service delivery from diverse standpoints. An emerging literature has begun describing the transition to virtual care during the pandemic and discussing the successes and challenges [36–40]. However, there is a paucity of information on patient experiences in general, and particularly on the experiences of youth receiving virtual MHSU care during the pandemic. This study builds on pre-pandemic research on virtual service delivery and emerging literature on virtual services during the pandemic by examining youth preferences for and experiences of MHSU services delivered virtually during COVID-19 pandemic.

## Objective

This paper examines youth preferences for and experiences of virtual MHSU services during the COVID-19 pandemic.

## Method

As part of a larger longitudinal study examining youth MHSU during the COVID-19 pandemic in clinical and non-clinical samples, this paper analyzes data from the August 2020 wave of data collection. Youth were defined as young people aged 14 to 29 [41].

### Participants

Four hundred nine participants completed the survey, including 164 drawn from a clinical sample and 245 from a non-clinical sample, representing 66% of the original sample. Details on the participant pools are described in Hawke et al. [7].

### Procedure

Participants received an email message with a web link taking them to an online survey on REDCap software [42], specifying that the study was about COVID-19 and mental health. After electronic written informed consent, youth completed a 20–30 min survey about MHSU experiences during the COVID-19 pandemic. Repeat survey questions were not carried out. As per our Research Ethics Board approval, consent was sought from all participants regardless of age, and parental consent was not sought. Data collection took place between August 7 and August 30, 2020, i.e., approximately 5 months after COVID-19 was declared a pandemic by the World Health Organization [43] and a state of emergency was declared provincially [44], and therefore about 5 months after youth MHSU services began moving to virtual modalities. Centre for Addiction and Mental Health (CAMH) Research Ethics Board approval was obtained.

### Youth engagement

Youth engagement is a core component of this study. As part of the CAMH McCain Centre Youth Engagement Initiative [45], youth co-researchers supported the study. In addition to regular consultations with youth during survey development, results were brought to youth co-researchers for discussion and interpretation purposes. The feedback of the youth co-researchers is presented in the Discussion section.

### Measures

Questions were developed by the research team, in consultation with local service providing agencies, to understand youth virtual MHSU service experiences. These were embedded into the larger survey. Youth co-researchers also collaborated on survey development, in line with the McCain youth engagement model [45]. Participants were asked about their service experiences, including whether they would consider and had experienced virtual MHSU services individually and, separately, in group format, and if so, what type of technology they had used. Based on their responses, they provided qualitative feedback on the types of services they would be interested in, why they might not consider such services, what the optimal features would be, and what the advantages and disadvantages of virtual services were.

The GAIN-Short Screener (GAIN-SS) is a self-report screener that identifies the likelihood of meeting diagnostic criteria for internalizing, externalizing and substance use disorders, as well as crime/violence issues [46]. Items are rated on a time-based scale for each symptom, ranging from zero (never) to four (past month). Three or more items endorsed at the past-month level suggest a high likelihood of meeting diagnostic criteria or requiring services in that area [46, 47]. The GAIN-SS is validated among youth [46].

### Analyses

Participants were included in the sample if they completed up to the end of the section querying about virtual services. Missing data was minimal, and frequencies are reported. The GAIN-SS Crime/Violence scale was not analyzed with service use preferences, given very low endorsement. Frequencies and chi-square tests were conducted to describe the sample and their virtual service experiences. After examining the demographics by clinical and non-clinical sample of origin, the two samples were merged to understand willingness to consider using virtual services and experience based on the presence of a MHSU concern. Willingness to consider and experience were then further examined across sample by age group (< 20, *N* = 134; vs. 20+, *N* = 271, 4 missing age) and pre-COVID-19 connection with services as reported by the participants at our first wave of data collection. The quantitative analysis was performed using the IBM SPSS statistics software (Version 25) [48].

A ‘conventional’ content analysis was conducted to analyze the qualitative data [49]. The limited research on youth perspectives of virtual care services during a pandemic made this approach most appropriate in analyzing the views of participants. The content analysis was performed by one of the authors (NYS), a female research analyst with a master’s degree in public health and training in health promotion. During the open coding process, a series of codes were identified, defined, and derived from data drawn from responses to each question [49]. In order to meaningfully group the codes, they were organized into categories based on similarities and differences. Confirmability of the results was established through team debriefings with NYS and the first author (LDH), a female scientist with a doctoral degree in psychology, to discuss the emergent categories. This process was repeated for each category until a team consensus was met. The qualitative analysis was performed on Microsoft Office Excel.

## Results

Table 1 presents the demographic and clinical characteristics of participants by sample. The non-clinical sample consisted of 245 participants aged *M* = 20.88 years (*SD* = 1.39). The clinical sample included 164 participants aged *M* = 20.25 (*SD* = 3.04), *t*(206) = 2.501, *p* = .013. Age ranged from 15 to 28 years. The clinical sample consisted of marginally more girls/young women than boys/young men, *χ*^*2*^(1) = 3.987, *p* = .046, φ = .101), and participants who were less likely to be employed prior to COVID-19 *χ*^*2*^(1) = 12.776, *p* < .001, φ = .183). The clinical sample was more likely to screen positive for internalizing, externalizing, or substance use disorders (*p*s < .001). There were no significant differences between samples regarding ethnicity, or Canadian-born, English speaking, or student status (*p*s > .10). While the univariate majority of the sample was a girl/young woman, Caucasian, student, and born in Canada, with English as a first language, this multivariate profile accounted for 50 (12.2%) participants.

**Table 1:** Demographic and clinical characteristic of participants by sample

	Non-clinical		Clinical	
	*N*	%	*N*	%
Gender				
Man/boy	85	34.7%	39	23.8%
Woman/girl	156	63.7%	113	68.9%
Another gender	4	1.6%	12	7.3%
Ethnic origin/background				
Caucasian	141	57.6%	99	60.7%
Asian (East and Southeast)	39	15.9%	9	5.5%
South Asian	23	9.4%	8	4.9%
Black (African, Caribbean, North American)	14	5.7%	8	4.9%
Multiple	9	3.7%	22	13.5%
Indigenous	4	1.6%	2	1.2%
Another background	15	6.1%	15	9.2%
First language English	222	90.6%	154	93.9%
Born in Canada	216	88.2%	145	88.4%
Student status				
Full time	154	62.9%	92	56.1%
Part time	14	5.7%	15	9.1%
Not a student	77	31.4%	57	34.8%
Employment status				
Full time	56	23.0%	26	15.9%
Part time	102	42.0%	47	28.7%
Mental health and substance use				
GAIN-SS Internalizing	93	38.1%	109	67.7%
GAIN-SS Externalizing	44	18.0%	59	36.6%
GAIN-SS Substance use	5	2.0%	34	21.1%
GAIN-SS Crime/violence	0	0.0%	2	1.2%

### Willingness to consider virtual services

**Fig. 1:**
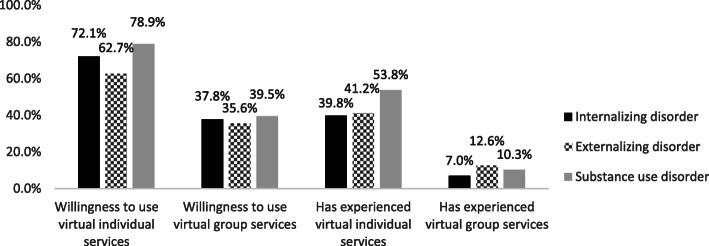
Proportion of participants who would consider and who have experienced virtual mental health and substance use services, among those who screen positive for an internalizing, externalizing or substance use disorder

The majority of participants screening positive for internalizing disorders (72.1%), externalizing disorders (62.7%) or substance use disorders (78.9%) would be willing to consider individual virtual services, but a minority would consider group virtual services (< 40% in each group, Fig. 1). Based on age (< 20, 20+), there was no difference in willingness for individual (62.8% for age < 20, 55.6% for age 20+, *χ*^*2*^(1) = 1.824, *p* = .177, φ = .068) or group virtual services (27.3% for age < 20, 29.3% for age 20+, *χ*^*2*^ = 0.158, *p* = .691, φ = .020). Among participants screening positive for at least one domain on the GAIN-SS, those who were already connected with MHSU services prior to the pandemic were significantly more likely to consider individual virtual services (77.4%) than those who were not already connected (63.0%, *χ*^*2*^(1) = 5.462, *p* = .019, φ = −.156). For group virtual services, these rates were 39.4 and 34.5% respectively, *χ*^*2*^(1) = 0.575, *p* = .448 φ = −.051.

### Experience of virtual services

39.8% of youth screening positive for an internalizing disorder have experienced individual virtual services, while 41.2% of those screening positive for an externalizing disorder and 53.8% of those screening positive for a substance use disorder have experienced individual virtual services (Fig. 1). Few youth screening positive in any domain have received group virtual services.

Among participants who have experienced individual virtual services, video format was reported by 70.2% of participants, phone by 61.2%, and chat by 18.2%. For group virtual services, video format was reported by 80.8% of participants, phone by 23.1%, and chat by 23.1%. Youth qualitatively described that they were currently accessing virtual counselling and therapy, medical and psychiatric services, and supplemental supports.

Among the 121 youth receiving individual virtual services, 86 (71.1%) screen positive for concerns in at least one domain on the GAIN-SS from among internalizing, externalizing and substance use disorders. Among the 26 youth receiving group virtual services, 18 (69.2%) screen positive in at least one domain. Based on age, there was no difference in whether youth had experienced group virtual services (4.7% for < 20, 7.4% for 20+, *χ*^*2*^ = 1.107, *p* = .293, φ = −.053), although younger youth were marginally more likely to have experienced individual virtual services (37.2% for age < 20, 27.0% for age 20+, *χ*^*2*^ = 4.321, *p* = .038, φ = .104).

### Perception of change

Among 144 participants who reported that MHSU services were in place for them prior to the pandemic, 67 (46.5%) reported that their services have changed for the worse during the pandemic. Another 25 (17.4%) considered their services to have not changed either for the better or for the worse. For 25 (17.4%), services are perceived to have changed for the better.

### Kinds of virtual activities

Qualitative findings are presented in Table 2. Participants expressed a desire for various forms of counselling or therapy and mindfulness/meditation, online supportive activities such as fitness, nutrition, and educational activities, as well as awareness initiatives. Some further pointed to the wish for recreational and fun activities.

**Table 2:** Summary of qualitative findings with exemplar quotes and associated codes

Question	Category	Quotes	General description
*Kinds of virtual activities wished for*	Online activities	Online workshops that provide information and advice on varying areas of concern related to mental health/wellness. Online chats with peers who are struggling with similar issues.	Activities such as fitness activities, educational activities, fun activities, interactive activities, surveys
	Awareness	Bringing awareness and spreading information about resources. Participating in studies like this one.	Various awareness initiatives
	Therapy	I would like one-on-one therapy to help me cope when I am in distress.	Counselling or therapy, skills-based, one-on-one therapy, mindfulness or meditation, family therapy
*Optimal features*	Platform	I think all formats would be beneficial in their own way, e.g., chats are good for people that find it hard to talk, or have a hard time making eye contact. Overall, though, video would seem to best.	Video call, phone, chat or text-based, online, in-person, discussion boards, smaller groups, any format
	Technological features	Clear and smooth audio and video, (un) muting audio and (dis) connecting video, option for sharing screens and files.	Smooth audio and video calls, option to share photos or files, convenience and ease, ability to turn off camera, mute button, option to share screens, good internet connection, optimal lighting, good camera quality
	Safety features	To be able to have conversations, in private via chat maybe so parents can’t listen in.	Privacy, maintain social distancing, anonymity features for video calls
	Care needs	Clear communication and a non-judgmental environment. Free virtual care for all.	Clear communication, non-judgmental environment, continuity, scheduled check-ups, free services
	Administrative	Consistent weekly and/or daily scheduling opportunities available.	Availability of appointment times, easy booking appointment
*Reasons for not considering*	Preference	I feel like mental health requires assistance in person. A human-to-human contact.	Do not care or feel like it, do not like the format, prefer to be in person
	No use for these services	I have done a lot of growing on my own with the help of family and [*clinician*]. The situation that brought me to [*services*] has been resolved and I feel like my mental health improved greatly with less worries now.	Already have supports in place, do not currently need them
	Discomfort	I would be embarrassed to talk about my issues with people who probably have more serious issues. I feel like I’m pretty privileged and should be able to figure it out.	Hesitant or too anxious to try, not comfortable with online, awkward
	Security & privacy concerns	As my family members are in close proximity within the house, I wish to use such services without any disruptions/in my own privacy (without anyone else being within earshot). Many are afraid of opening up about mental health in the first place. Group discussion virtually would increase anxiety for me because I cannot sense how the group is and what if they are recording what we are saying/others in the room are hearing it. There would be even less confidentiality in my opinion.	Security concerns, privacy issues with living at home, privacy issues in general
	Technology	Would rather speak in person if anything. Don’t feel comfortable being online and my internet is also not strong enough to support that.	Poor internet connection
*Advantages of virtual services*	Convenience and accessibility	Convenience. No need for an app or anything else. I just called my school psychologist, and we got to talking - which helped me a lot. There was nothing complicated about it.	No travel, convenience, uncomplicated, accessible services, flexible scheduling, able to receive care during a pandemic, fast, more inclusive
	Comfort	I can avoid the embarrassment of crying in person.	Feeling comfortable with online services, able to express feelings virtually, more comfortable with phone conversations
	Practicality	Talking to someone responsible and smart is always helpful. I keep track of the things I do and where I am at during the week and bouncing what I am up to off of a social worker can go along way when it comes to maintaining a healthy pathway.	Good for communication, useful, cost-effective, helped mental health, helpful, ability to read what was previously expressed, showed up to all appointments, worked
	Human connection	Being able to see the person I was talking to as opposed to just speaking to them on the phone.	Seeing their faces, sense of community, feeling supported
	Safety	Able to get the support I need while staying safe, and while keeping therapist/social workers safe.	Socially distanced, safe, feeling more secure, privacy
*Disadvantages of virtual services*	Lack of human connection	It’s very impersonal. I can’t connect with the person and I tend to forget what people tell me when talking on the phone.	Less personal (e.g., no human connection), not in-person, feeling inauthentic over phone, feelings of loneliness, less personalized care, longer establishing rapport
	Technology	Potential Wi-Fi issues, and it’s hard to find a spot with okay lighting and sound.	Technological limitations, poor internet connection, not always reliable, video use
	Privacy, security, safety	In my home, it’s extremely difficult to have a real sense of privacy (no matter which room you’re in someone can here you or is waiting to use it).	Lack of privacy at home, security concerns
	Emotional barriers	I kind of quit therapy because I became unmotivated to pick up the phone and my psychiatrist’s office always messes up and doesn’t send me the link to our meeting so those get skipped.	More distractions, harder to focus, forgetful, unmotivated
	Discomfort	Sometimes it is more difficult to talk about negative and painful things that have happened over the phone.	Does not feel comfortable, awkward, difficult to discuss painful experiences over phone, unfamiliar
	Administrative issues	Not always reliable if professionals are unavailable. Unlikely to receive consistent care since you talk to different people.	Longer time between appointments, unreliable scheduling
	Structure	I don’t have to leave the house/get changed/wake up early (I’m completely out of whack; I don’t have a system, I’m not living life as it normally is).	Time consuming, lack of routine
	Poor experience	It was super boring, isolating, and I felt like I was a horrible person because of how they treated me.	Did not receive care needed

### Optimal features of virtual care

For individual virtual care, video calls received the largest proportion of responses, followed by phone or voice calls and text or chat-based support. Some youth were accepting of any format. Technical features included smooth audio and video, good internet connection and lighting, good camera quality, a mute button and the ability to turn off the camera and share files. Youth wanted clear communication in a non-judgemental environment. Many youth valued anonymity and privacy. They wanted services to be free, continuous, convenient and accessible in terms of booking and availability.

In terms of group virtual services, the largest proportion of participants wanted video calls, followed by phone and chat/text. Technical features such as a mute button, good camera quality, the ability to turn off the camera and share files were also appreciated. Some youth specifically reported wanting to access group services, while others reported a preference for one-on-one services.

### Reasons for not considering

Participants who were not willing to consider individual virtual services expressed a preference for in-person services, mostly for human connectedness and more personal care. Others expressed anxiety or discomfort with using virtual platforms, and security, privacy (e.g.*,* living with at home), and technical concerns (e.g.*,* poor internet connection). Many would not consider virtual services because they did not need them or already had supports in place. These findings were consistent across group virtual services, with one added finding for group virtual services: youth expressed that they preferred individual services.

### Advantages and disadvantages of individual of virtual services

Participants who had received individual virtual services qualitatively reported service accessibility advantages including convenience, easy scheduling, and the lack of travel time. They appreciated being able to connect with a professional safely in a comfortable, socially distanced manner during COVID-19, and appreciated seeing the service provider’s face. Some participants further reported that virtual individual care was good for communication, an uncomplicated process, and helped their mental health.

Expressed disadvantages of individual virtual care included discomfort and problematic interaction factors: it was less personal and awkward, and it was difficult to read body language, establish rapport, and discuss sensitive issues. Participants mentioned privacy concerns and noted greater distractions and interruptions, with more challenges focusing. They expressed technological issues, including a poor internet connection and security concerns. Many did not like that it was not in person. Some further pointed to administrative challenges around scheduling.

### Advantages and disadvantages of group virtual services

Participants who had received group virtual services qualitatively mentioned accessibility as an advantage, including convenience and simplicity of use. They appreciated the human connection. Some further appreciated the lack of travel time, comfort of remote services, and flexible scheduling. They appreciated the ability to receive care during the pandemic. Disadvantages were less reported on, although participants did state that there was less human connection, it was less personal, and it was harder to focus. They further noted privacy concerns and technological limitations, such as a poor internet connection. Some mentioned the fact that it is not in person as a disadvantage.

## Discussion

This study examined youth attitudes toward and experiences of virtual services for MHSU during the COVID-19 pandemic, providing important youth perspectives on virtual MHSU services during an unprecedented rapid move toward virtual service delivery. The majority of youth screening positive for MHSU challenges would consider using individual virtual MHSU services, but fewer are willing to use group services. While some have experienced such services, using a variety of modalities, many youth with MHSU challenges have not. Overall, virtual service delivery has a number of advantages, but also multiple disadvantages.

Previous studies have shown that youth are willing to use virtual MHSU services and that some prefer virtual services [50]. Similar advantages and disadvantages of virtual care have been reported by youth and clinicians alike [26, 50–55]. For example, accessibility benefits have been identified [26, 50, 55], as has the value for interpersonal connectedness in virtual services [54]. Confidentiality concerns have been previously expressed [56–59], particularly when accessing services in the home [58]. Texting or chatting modalities might reduce this challenge for some youth [58]. Some youth expressed discomfort regarding virtual services as a whole; it may be that in-person services are required for a subset of youth, or it may be possible to work with these youth to understand how to increase comfort with virtual services. Importantly, different youth have different preferences, and diversity of service options should therefore be available.

The digital determinants of health are more important than ever as they appear to be exacerbating health problems during COVID-19 [60, 61]. Concerns have been raised around inequitable access to digital health services [32]. Although remote service provides physically distanced care during a pandemic, future research should consider strategies that may address the health inequities exacerbated by the shift to virtual MHSU services, for those without adequate technology and a safe, private space to use it.

From a stakeholder-engaged perspective, we worked directly with youth members of the McCain Centre Youth Engagement Initiative (YEI) [45] to develop the study and interpret the findings. Youth staff agreed that the findings resonated with them and the perspectives they are hearing from other Canadian youth. They highlighted the willingness to consider virtual services among youth who are already connected to MHSU services, but less so among those who are not already connected to services. They emphasized accessibility benefits and hoped that these benefits would persist after the pandemic. They also noted concerns around in-home confidentiality and technological barriers, especially for youth without easy access to technology. They added that cameras make some youth self-conscious and can provide a very personal look into a youth’s private space. They further indicated that virtual care does not provide a needed psychological separation between the therapeutic environment and the home environment. The youth importantly noted that youth are a heterogeneous group, emphasizing the importance of offering a variety of services and modalities to meet the needs of different youth.

While the move to virtual services was conducted as an urgent response to a public health emergency, it is likely that some of the changes will be sustained [53]. As a new paradigm of virtual MHSU service delivery emerges, it will be essential to rigorously evaluate virtual interventions for efficacy and effectiveness, to understand for whom virtual care has benefits, and examine virtual interventions that can be implemented effectively into existing clinical service systems. Adapting virtual services requires a clear understanding of youth experiences and preferences [62]. Sustained efforts to ensure the diverse needs of youth are met are thus essential in the era of digital health.

### Limitations

The survey was limited to Ontario, Canada; a more diverse, representative sample collected over a broader geographical area would increase generalizability. Qualitative findings are derived from short survey answers rather than semi-structured interviews and are therefore limited in breadth. Since the survey was conducted online, it missed youth who do not use the internet regularly. Response rates may be differentially associated with level of MHSU challenges. Future research should connect with youth who are not regular internet users or who have limited technology access to understand their perspectives on virtual services, perhaps including telephone alternatives, and on possible means of mitigating technological barriers.

## Conclusions

As the system continues expands on virtual models of MHSU service delivery, it is essential that researchers, service providers, and interventionists engage directly with youth, caregivers, and service providers [33, 34, 45, 63, 64] to understand their perspectives and experiences. While the majority of youth are willing to consider virtual services, many youth have not received virtual services despite willingness and clinical need. Diverse, identifiable, accessible, technologically stable virtual services are required to meet the needs of different youth, possibly with in-person options for some youth. Future research, engaging youth in the research process, is needed to evaluate the efficacy of the virtual service modalities.

## Data Availability

Data are available upon reasonable request to the corresponding author (Joanna.Henderson@camh.ca), with Research Ethics Board approval.

## Abbreviations

MHSU: Mental Health and Substance Use
GAIN-SS: GAIN Short Screener
